# Risk factors for community-acquired *Escherichia coli* bacteraemia: a systematic review protocol

**DOI:** 10.12688/wellcomeopenres.14804.1

**Published:** 2018-09-19

**Authors:** Anna Aryee, Suvi Härmälä, Laura Shallcross, Andrew Hayward

**Affiliations:** 1 Institute of Health Informatics, University College London, London, NW1 2DA, UK; 2 Institute of Epidemiology & Health Care, University College London, London, WC1E 6BT, UK

**Keywords:** Escherichia coli bacteraemia, community acquired, risk factors, antimicrobial resistance

## Abstract

**Introduction: **Rates of community-acquired *Escherichia coli* bacteraemia (ECB) have been consistently rising. As rates of antimicrobial resistance (AMR), particularly in Gram-negative bacteria, are also increasing, this is of concern both for management of individual patients and healthcare systems. There is currently little data on the risk factors for development of community-acquired ECB: this review aims to identify these risk factors in order to inform community interventions to reduce ECB as well as antibiotic prescribing policy.

**Methods and analysis: **We will search Medline (Ovid), Embase (Ovid), Web of Science/Scopus and the Cochrane Central Register of Controlled Trials for published reports on observational and experimental primary research studies involving patients admitted to hospital with community-acquired ECB. Two reviewers will independently screen the studies for eligibility, perform data collection and assess study quality and risk of bias. Random effects meta-analyses will be performed if appropriate.

**Ethics and dissemination: **No primary data will be collected for this study and so formal ethical approval is not required. We will publish the results of our review in relevant peer-reviewed medical journals, and will also seek to present them at relevant medical conferences.

**PROSPERO registration number:** CRD42018104402

## Introduction

### Background

*Escherichia coli* is the commonest organism to be isolated in blood cultures in the UK and elsewhere in Europe ^1– 3^. Whilst rates of MRSA bacteraemia have been decreasing over the past few years, *Escherichia coli* bacteraemia (ECB) has been consistently increasing ^3, 4^. This is a worrying phenomenon, as antimicrobial resistance (AMR) in *Escherichia coli* and other Enterobacteriaceae makes invasive infections progressively more difficult to treat ^5^. Whilst an infection in any part of the body has the potential to cause a bloodstream infection, the majority (approximately 50%) of ECB are from a urinary source ^1, 4^. Other sources include an infection in the gastrointestinal or biliary tract, and less commonly respiratory tract infection. A small proportion of ECB also has an unidentified source ^1^.

Rates of ECB are highest in young children and the elderly, and have been shown to vary with the seasons, in a way which has not been identified in, for example, MRSA bacteraemia ^6^. The risk factors for ECB are not well described. As most ECB is community acquired, it has been argued that there is limited scope for preventative strategies. Recent studies, however, show that a large proportion of community-acquired cases are healthcare associated, with patients having had contact with hospital or outpatient services ^7^. This potentially increases the scope for interventions that could reduce their incidence.

### Rationale for the review

Rates of AMR are rising globally and are of great concern to clinicians and policy-makers as they are associated with higher morbidity and mortality, longer healthcare stays and higher healthcare costs ^8, 9^. In light of this, the trend for increasing rates of ECB is troubling. Given that most ECB is community-acquired, any interventions to reduce these rates must be based on robust evidence of the risk factors contributing to its acquisition. AMR is driven by antibacterial use, whether this is appropriate or inappropriate, and judicious antibacterial use must be based on information on which patients are more or less likely to suffer a severe outcome from their infection.

To our knowledge, no systematic review to date has investigated the risk factors for community-acquired ECB. This review therefore aims to provide a systematic synthesis of the available published evidence. The results may inform community interventions to reduce ECB, as well as inform antibiotic prescribing policy.

### Objectives

The objective of this review is to investigate the risk factors for developing community-acquired *Escherichia coli* bacteraemia in patients of all ages in high income countries (as defined by the World Bank).

## Protocol

This study protocol follows the recommendations by the Preferred Reporting Items for Systematic Review and Meta-Analysis Protocols (PRISMA-P) 2015 ^10^. A completed PRISMA-P checklist can be found in Supplementary File 1.

### Eligibility criteria

***Study designs.*** Observational and experimental primary research studies will be eligible for inclusion. The following study designs will be considered: cohort, case-control and cross-sectional studies; randomised and non-randomised, controlled and non-controlled trials; reviews and meta-analyses (as a means of identifying the source studies). Only studies which include a control group who did not have community-acquired ECB will be included. We will exclude case reports, case series, opinion papers, letters to the editor, policy papers, conference proceedings, comments and study protocols without baseline data.

***Participants.*** We will include participants of all ages who were admitted to hospital with community-acquired ECB.

***Exposures.*** There will be no restrictions on the types of exposures (risk factors) considered in the primary studies, and studies will be considered provided they include quantitative data on the risk factors for community-acquired ECB. Potential risk factors may include clinical features, demographic characteristics, dehydration, presence of a urinary catheter, prior hospital contact, prior antibiotic usage and co-morbidities such as diabetes mellitus and chronic kidney disease.

***Comparators.*** The main comparator will be participants without bacteraemia. However, as we are aiming to include a broad range of studies, control groups may include participants with hospital-acquired ECB and participants with community-acquired bacteraemia due to another organism(s). A control group of participants with hospital-acquired ECB will allow us to identify risk factors specifically associated with acquisition of ECB in the community – potentially leading to interventions in primary care or public health campaigns. A control group of participants with community-acquired bacteraemia due to another organism will allow us to identify risk factors specifically associated with *Escherichia coli* itself, potentially informing antibiotic treatment strategies.

Studies which assess particular clinical conditions, for example malignancy, in relation to community-acquired ECB will be included if they also include a control group which does not have the condition of interest. Studies comparing one antibiotic regimen against another in treating community-acquired ECB will also be included if they fit the other eligibility criteria.

***Outcome measures.*** The outcome will be community-acquired ECB, as defined by a blood culture which is positive for *Escherichia coli* within +/- 1 day of admission to hospital.

***Time frame.*** There will be no restriction by duration of follow-up or date of publication.

***Setting.*** Only studies carried out in high income countries (as defined by the World Bank) will be included ^11^.

***Language.*** Only studies published in English will be included.

### Information sources

The following databases will be searched: Medline, Embase, Web of Science/Scopus and the Cochrane database. Each database will be searched separately and the search strategy first developed in Medline will be adapted to each database interface as appropriate. Relevant studies from the reference lists of the eligible studies identified through the electronic searches will also be included.

### Search strategy

The above databases will be searched for the above dates for relevant studies. The literature search will use the following terms (with synonyms and closely related words): “ *Escherichia coli*” AND “bacteraemia” AND “community-acquired infections”. The searches will not be limited by study design, but will be limited to those undertaken in high-income countries (as defined by the World Bank) and published in English ^11^. The search strategy for Medline is outlined in Table 1. The full list of sources and search strategies used can be found in Supplementary File 2.

**Table 1.** Preliminary Ovid (Medline and Embase) search strategy.

Search concept	Search terms
*Escherichia coli*	1. Escherichia coli/
	2. E* adj coli.mp
	3. 1 or 2
Bacteraemia	4. BACTEREMIA/
	5. Bacter*mia.mp
	6. (bloodstream adj3 infection*).mp
	7. Blood-borne Pathogens/
	8. Septic*mia.mp
	9. (Blood* adj3 (pathogen* or infection* or bacteri* or microbe* or microbial*)).mp
	10. Blood Culture/
	11. (blood adj culture).mp
	12. 4 or 5 or 6 or 7 or 8 or 9 or 10 or 11
Community-acquired infections	13. Community-Acquired Infections/
	14. (community*acquired adj infection*).mp
	15. (community-acquired adj5 healthcare-associated).mp
	16. (community acquired adj5 healthcare associated).mp
	17. Primary Health Care/
	18. (primary adj (health*care or care).mp
	19. General Practice/
	20. (general adj practice).mp
	21. Family Practice/
	22. (family adj practice).mp
	23. 13 or 14 or 15 or 16 or 17 or 18 or 19 or 20 or 21 or 22
	24. 3 and 12 and 23

### Study records: data management, selection process, data collection process

The search results will be uploaded into the Mendeley reference management software, and duplicate records will be removed. The study records will then be uploaded into DistillerSR, a web-based systematic review management software. Studies will be screened for eligibility by two independent reviewers (A.A. and S.H.). Data will be extracted from the reports using specifically designed data extraction forms which will be piloted prior to use. Data will be extracted independently and in duplicate by the two reviewers. Discrepancies will be discussed with a third reviewer (L.S.) and agreed by consensus. Where necessary, clarification will be sought from study investigators. The study selection process will be recorded and presented in flow diagram format according to the recommendations of PRISMA.

### Data items

Data will be sought for the following variables:

• Study characteristics (design, location, year of recruitment)
• Study participants: inclusion and exclusion criteria, method of recruitment/selection, study population characteristics (age, gender, socioeconomic group, co-morbidities, residential/nursing home resident)
• Identified exposures (risk factors) e.g. urinary catheter use, interventional procedures, dehydration, prior admissions to hospital, prior/recurrent UTI, pregnancy
• Bacteraemia data: date of blood culture in relation to admission, antibiotic sensitivities of *Escherichia coli* isolate, source of bacteraemia (urinary, GI/biliary, respiratory, cardiovascular, unknown/unspecified)
• Interventions and comparators: antibiotic regimen and duration, level of care (general ward/ICU/outpatient treatment), follow-up time.

### Outcomes and prioritisation

The only outcome of interest for this systematic review is community-acquired ECB. Multiple exposures will, however, be considered, with potentially modifiable exposures, for example urinary catheter use and dehydration, taking priority.

### Risk of bias in this review and individual studies

We will conduct the systematic review in accordance with this protocol, and any differences between the methods of the complete review and this protocol will be reported in the review.

Risk of bias will be assessed using the Newcastle-Ottawa scale for non-randomised studies, and the Cochrane Risk of Bias Tool for randomised controlled trials ^12, 13^. Each study will be independently assessed by A.A. and S.H. for selection, performance, attrition and reporting bias, and disagreements will be resolved by discussion and consensus. If consensus cannot be reached, a third reviewer (L.S.) will be consulted to adjudicate.

### Data synthesis

Formal meta-analysis will be carried out only if we identify two or more studies which we consider homogenous in terms of clinical characteristics, study design and methods. In this case we will synthesise the available information using random effects meta-analysis, and report on factors positively or negatively associated with development of ECB using risk ratios, odds ratios or rate ratios (depending on study design) with 95% confidence intervals. If there are insufficient studies for meta-analysis, we will synthesise the data narratively. The outcomes will be analysed at the level of individual study participants for each study, and we will attempt to obtain any missing numerical outcome data by contacting investigators directly. We will explore the impact of including studies with high levels of missing outcome data on the measure of association in sensitivity analyses. We will assess heterogeneity between studies by presenting a forest plot of the review outcome, and will then calculate the formal heterogeneity variance statistics τ ^2^, I ^2^ and the Q-statistic. Heterogeneity will be considered as substantial if the τ ^2^ is greater than 0, I ^2^ is more than 30% and the P value for the Q-statistic is less than 0.10.

### Assessment of publication bias

We will assess publication bias by visual inspection of funnel plots.

### Assessment of strength of evidence

We will use the Grading of Recommendations, Assessment, Development and Evaluation (GRADE) framework to assess the strength of the body of evidence for this systematic review ^14^.

### Ethics and dissemination

No primary data will be collected for this study and so formal ethical approval is not required. We will publish the results of our review in relevant peer-reviewed medical journals, and will also seek to present them at relevant medical conferences.

## Data availability

No data are associated with this article.

## Supplementary material

Supplementary File 1. Completed PRISMA-P checklist.

Supplementary File 2. Search strategies for Web of Science and Cochrane Database.
