# Supplementary material for: Risk factors for community-acquired Escherichia coli bacteraemia: a systematic review protocol
Source: Wellcome Open Res. 2018 Sep 19;3:117. [Version 1] doi: 10.12688/wellcomeopenres.14804.1 (PMC6758834; doi:10.12688/wellcomeopenres.14804.1)
Supplement: Supplementary file 2 [file wellcomeopenres-3-16132-s0001.tgz › 478f22c1-c32d-447a-9ffc-9d9fe535f4fc_Supplementary_file_2.docx]

**Supplementary file 2**

Table 1. Preliminary Web of Science search strategy

| **Search concept** | **Search terms** |
| --- | --- |
| *Escherichia coli* | 1. TS=“E* coli” |
| Bacteraemia | 1. TS=Bacter*mia |
|  | 1. TS=(Bloodstream NEAR/3 infection*) |
|  | 1. TS=Septic*mia |
|  | 1. TS=(Blood* NEAR/3 (pathogen* or infection* or bacteri* or microbe* or microbial*)) |
|  | 1. TS=“Blood culture” |
|  | 1. 2 or 3 or 4 or 5 or 6 |
| Community-acquired infections | 1. TS=“Community*acquired infection*” |
|  | 1. TS=(Community acquired SAME healthcare associated) |
|  | 1. TS=“Primary health*care” |
|  | 1. TS=”Primary care” |
|  | 1. TS=“General practice” |
|  | 1. TS=“Family practice” |
|  | 1. 8 or 9 or 10 or 11 or 12 or 13 |
|  | 1. 1 and 7 and 14 |

Table 2. Preliminary Cochrane Database search strategy

| **Search concept** | **Search terms** |
| --- | --- |
| *Escherichia coli* | 1. MeSH term: [Escherichia coli] explode all trees |
|  | 1. “E* coli” |
|  | 1. 1 or 2 |
| Bacteraemia | 1. MeSH term: [Bacteremia] explode all trees |
|  | 1. Bacter*mia |
|  | 1. Bloodstream near/3 infection* |
|  | 1. MeSH term: [Blood-Borne Pathogens] explode all trees |
|  | 1. Septic*mia |
|  | 1. Blood* near/3 (pathogen* or infection* or bacteri* or microbe* or microbial*) |
|  | 1. MeSH term: [Blood Culture] explode all trees |
|  | 1. “Blood culture” |
|  | 1. 4 or 5 or 6 or 7 or 8 or 9 or 10 or 11 |
| Community-acquired infections | 1. MeSH term: [Community-Acquired Infections] explode all trees |
|  | 1. “Community*acquired infection” |
|  | 1. Community*acquired near/5 healthcare*associated |
|  | 1. MeSH term: [Primary Health Care] explode all trees |
|  | 1. “Primary health*care or “primary care” |
|  | 1. MeSH term: [General Practice] explode all trees |
|  | 1. “General practice” |
|  | 1. MeSH term: [Family Practice] explode all trees |
|  | 1. “Family practice” |
|  | 1. 13 or 14 or 15 or 16 or 17 or 18 or 19 or 20 or 21 |
|  | 1. 3 and 12 and 22 |
