# Supplementary material for: Risk factors for community-acquired Escherichia coli bacteraemia: a systematic review protocol
Source: Wellcome Open Res. 2018 Sep 19;3:117. [Version 1] doi: 10.12688/wellcomeopenres.14804.1 (PMC6758834; doi:10.12688/wellcomeopenres.14804.1)
Supplement: Peer review history

# Peer review: Reviewer response for version 1

**DOI:** 10.21956/wellcomeopenres.16132.r36308

**Published:** 2019-09-23

**Authors:** Céire Costelloe

**Affiliations:** 1 Faculty of Medicine, School of Public Health, Imperial College London, London, UK

- The research aims to systematically review the literature on risk factors for acquisition of CA E. coli bacteraemia in high income countries. The protocol is well described, adheres to PRISMA-P guidelines and includes both experimental and non-experimental designs, essential for inclusion in this research.
- The outcome measure is conservative in terms of CA definition as +/-1 day of admission, rather than 48 hours. The authors may find that more studies report CA based on +/- 2 days of admission.
- Details on a proposed meta-analysis are included, to be performed contingent on data availability, which is appropriate.
- The research will add to the body of evidence on risk factors for E. coli bacteraemia. 

I confirm that I have read this submission and believe that I have an appropriate level of expertise to confirm that it is of an acceptable scientific standard.

# Peer review: Reviewer response for version 1

**DOI:** 10.21956/wellcomeopenres.16132.r36085

**Published:** 2019-08-08

**Authors:** Mark Melzer

**Affiliations:** 1 Infectious Diseases Department, Royal London Hospital, Barts Health NHS Trust, London, UK

It is an important area of research as the main effort to date has been focused on hospital-acquired *E. coli* bacteraemia.

 When reviewing the literature the authors should focus upon the following risks (potentially mitigated) which are potentially relevant for their community study:

- Hydration work, particularly in nursing homes and other healthcare facilities.
- Public health messages such as personal hygiene (e.g. wiping front to back) and pre/post voiding following intercourse.
- The effect of GP prescribing more nitrofurantoin and less trimethoprim for treatment of lower UTIs, potentially preventing more hospital admissions caused by pyelonephritis/ *E. coli* bacteraemia.
- The effect of prophylaxis of recurrent UTIs, both antibiotic and non-antibiotic, again preventing pyelonephritis/bacteraemia.
- Community onset *E. coli* bacteraemias related to problems around long term urinary catheters (e.g. blockage/difficult changes in community). It would be interesting to examine the hypothesis that catheter passports and access to good community continence services reduce these problems. Also, should more patients have supra-pubic catheters vs urethral catheters (or even intermittently catheterise to reduce the risk of infection/ *E. coli* bacteraemia)?

I confirm that I have read this submission and believe that I have an appropriate level of expertise to confirm that it is of an acceptable scientific standard.
